# LONG-TERM BENEFITS OF A TAILORED STRENGTH TRAINING INTERVENTION ON ARM FUNCTION IN CHRONIC STROKE SURVIVORS: A FOLLOW-UP STUDY

**DOI:** 10.2340/jrm-cc.v8.42941

**Published:** 2025-03-24

**Authors:** Marie-Hélène MILOT, Stephania PALIMERIS, Yavuz SHAHZAD, Hélène CORRIVEAU, François TREMBLAY, Marie-Hélène BOUDRIAS

**Affiliations:** 1 Faculté de médecine et des sciences de la santé, Université de Sherbrooke, École de réadaptation, Sherbrooke, Québec, Canada; 2 Centre de recherche sur le vieillissement, CIUSSS de l’Estrie-CHUS, Sherbrooke, Québec, Canada; 3 Faculty of Medicine and Health Sciences, School of Physical and Occupational Therapy, McGill University, Montréal, Québec, Canada; 4 BRAIN Lab, Jewish Rehabilitation Hospital, Laval, Québec, Canada; 5 Montreal Center for Interdisciplinary Research in Rehabilitation (CRIR) and CISSS-Laval, Montréal, Québec, Canada; 6 Bruyère Research Institute, Ottawa, Ontario, Canada; 7 Faculty of Health Sciences, School of Rehabilitation Sciences, University of Ottawa, Ottawa, Ontario, Canada

**Keywords:** stroke, arm function, strengthening exercises, motor evoked potential, follow-up evaluation

## Abstract

**Objective:**

We showed that a tailored strengthening intervention based on the size of motor evoked potentials (MEPs) in the affected arm was effective in improving function in chronic stroke survivors. Here, we investigated whether the short-term gains in arm function were maintained at 1-year follow-up.

**Subjects:**

Twenty-five participants at the chronic stage of a stroke.

**Methods:**

Participants were classified in the light (LI; MEPs 50–120 μV, *n* = 8) and high (HI; MEPs > 120μV, *n* = 17) intensity training groups. The strengthening protocol consisted of adjusted exercises for the affected arm (3X/week; 4 weeks). The Fugl-Meyer Stroke Assessment (FMA), Grip strength (GS) and Box and Block test (BBT) were assessed at baseline, post-intervention and at 1-year follow-up. Changes in clinical measures were compared using repeated-measures ANOVA.

**Results:**

A significant effect of time was noted on all outcome measures [FMA: *p* < 0.001; BBT: *p* = 0.05; GS: *p* < 0.001], but the LI group improved more on the FMA (*p* = 0.003) and maintained their gains at 1-year follow-up (*p* = 0.527) than the HI group.

**Conclusion:**

The size of MEPs in the affected arm could be a significant factor in influencing responses to strengthening exercises post-stroke and allow gains to be maintained up to 1 year post-intervention.

## LAY ABSTRACT

Following a stroke, adjusting a training program on the integrity of the brain’s motor pathway allowing arm movement translates into short-term gains in arm function. The aim of this study was to assess whether the gains observed were maintained for up to 1 year following training. Thus, 25 participants were assigned to a low or a high-intensity training group, depending on the integrity of their brain’s motor pathway. Each participant trained his affected arm 3/X a week for 4 weeks. Function of the affected arm was assessed before and after training as well as 1 year following the end of the training. Results showed that training allowed significant gains in arm function, but the participants in the low-intensity training group improved better. Adjusting a training protocol to each individual’s integrity of his brain’s motor pathway allows for gain in arm function that is maintained up to 1 year post-training.

Stroke is the third-leading cause of disability worldwide (1). Paresis of the upper extremity (UE) contralesional to the affected brain areas is among the most common consequences of stroke, and persists at the chronic stage of a stroke (> 6 months post-stroke) (2). UE paresis can impede the ability of individuals to accomplish basic activities of daily living (e.g. eating, grooming), as well as instrumental activities of daily living (e.g. shopping, housekeeping) (3–5). For example, at 1 year post-stroke, because of UE paresis, 33% of individuals require assistance for dressing the upper body, and more than 75% require full assistance for meal preparation (3). This decrease in UE performance also translates into a decrease in social participation and a poorer level of satisfaction from life (3, 4).

Strengthening interventions have been shown to be effective in promoting neuroplasticity (6), motor capabilities (7–12), and strength (6, 7, 10, 13) in acute and subacute stroke survivors. For example, Fang and colleagues reported significant improvement in UE motor impairment in participants in the acute phase of stroke who received 45 min of daily supervised physiotherapy for 4 weeks compared to those without physical therapy (14). These findings in the acute phase are consistent with the notion that the first 3 months are a critical window for neuroplasticity and neural reorganization (15), which also corresponds to the time when most of the recovery is seen post-stroke (16).

There is growing evidence that intense rehabilitation interventions can also reduce motor impairments in chronic stroke survivors (6, 8). For instance, Beaulieu and colleagues investigated the effect of a resistance training intervention, paired or not with transcranial direct current stimulation (tDCS), in a group of 14 chronic stroke survivors (8). The intervention consisted of 60 min of exercises, 3 times per week for 4 weeks, targeting the affected UE. Although using tDCS did not lead to additional functional UE gains, both groups showed improvement in response to progressive resistance exercise. Recently, our group investigated the effect of a strengthening intervention targeting the UE in a large sample of chronic stroke survivors (*n* = 90) (17, 18). Participants were stratified into 3 intensity groups based on the size of motor evoked potentials (MEPs) elicited by transcranial magnetic stimulation (TMS) in the affected hand, which provided an index of corticospinal integrity and potential responsivity to training. Our results showed that adjusting the training intensity based on MEP size led to clinically significant gains in the affected UE for all participants, regardless of baseline stroke severity (17, 18).

There are still controversies as to whether the improvements gained from exercise interventions have long-term benefits in post-stroke survivors (19–27). Wu and colleagues compared the long-term recovery trajectories for 2 types of intervention: robot-assisted therapy and intensive training aiming to match robot-assisted therapy, which were compared with usual care (26). Both intervention groups underwent 1-h functional UE supervised training 3 times per week for 12 weeks until about 36 sessions were completed. Post-training, those in the intervention groups demonstrated greater improvement in UE function relative to the usual care group; however, at follow-up 36 weeks, no difference was detected. On the other hand, Stinear and colleagues investigated the effects of a 30-day training program on participants in the chronic stage and showed that gains in UE function were maintained up to 3 years post-intervention. Interestingly, the participants who maintained their gains also exhibited MEPs in response to TMS (27), indicating some preservation of corticospinal integrity. As stated earlier, our own investigation provided further evidence that the presence of MEPs is indeed a critical factor influencing the response to exercises in the affected UE (17, 18).

Here, our goal was to describe our observations collected from a subset of participants who completed our MEP-based strengthening intervention and were reassessed at 1-year follow-up. Based on studies that have found sustained long-term gains in function with exercise in chronic stroke survivors (21, 22, 24, 25, 27), we expected that the short-term gains in UE function post-training would still be detectable in the long term.

## METHODS

A detailed description of the study’s protocol and entry criteria is given elsewhere (17). In brief, participants at the chronic stage of a stroke were allocated to 3 training intensity groups based on the size of MEPs (peak-to-peak amplitude) elicited by supramaximal TMS pulses (1.3 X motor threshold) applied over the hand motor area of the lesioned hemisphere using the first dorsal interosseous (FDI) as the target muscle. Participants with MEPs < 50 μV were allocated to a low-intensity (LI) group, those with MEPs between 50 and 120 μV to a moderate-intensity (MI) group and those with MEPs > 120 μV to a high-intensity (HI) group. In each training group, the strength training program consisted of lifting dead weights, specifically targeting the shoulder, elbow flexors, and wrist extensors of the affected arm. At the beginning of each week of training, participants’ 10 RM (the maximal load that could be lifted 10 times consecutively) was assessed to estimate their 1 RM (28). The estimated 1 RM was then used to calculate the baseline at which participants began the upcoming week of training: those in the LI group began at 35% of their 1 RM, while MI and HI participants began at 50% and 70%, respectively. The intensity of training was increased by 5% weekly for the duration of the intervention so that at the end of the 4 weeks, participants in the LI group trained at 50% 1 RM, while the MI and HI groups, respectively, trained at 65% and 85% 1RM. The training also targeted grip strength (GS) using the Jamar® hydraulic hand dynamometer. The strengthening protocol also included anodal tDCS of the affected motor area (2 mA, 20 min) while participants performed their exercises. However, since no difference was detected in the clinical outcomes between sham and real tDCS in our group of participants (see Palmeris et al. 18), this aspect is not discussed here.

**Fig. 1:**
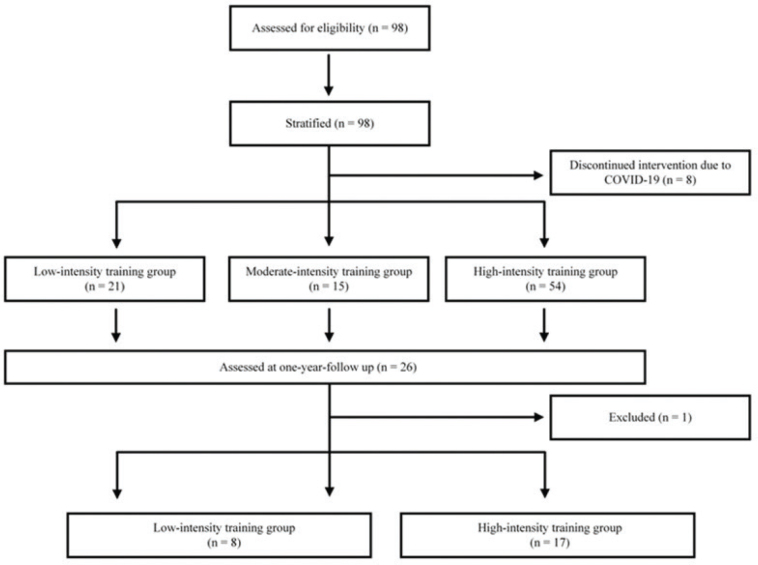
Study flow diagram.

All participants underwent clinical assessment of their UE by an experienced physiotherapist who was blinded regarding group allocation. The assessment took place in each site laboratory setting and lasted about 90 min. The assessment was performed at 3 time points: at baseline, prior to training (T1), immediately after the intervention (T2), and at 1-year follow-up (T3). The assessment included the following primary outcome measures: (*i*) the UE-FMA (29), (*ii*) the Box and Block test (BBT) (30), and (*iii*) GS, measured in kg (average of 3 trials). Several secondary outcome measures were also considered, including a self-reported quantity and quality of use of the paretic UE, quantified by the Motor Activity Log (MAL) (31), and the active range of motion (AROM) in flexion at the affected shoulder, elbow, and wrist (Fig. 1). All these tests are valid and present good psychometric properties for individuals with a stroke (32–35).

Prior to taking part in this study, all participants signed a consent form approved by the Research Ethics Committee (REC) of the CIUSSS de l’Estrie-CHUS (MP-22-2016-630) and Bruyère Research Ethics Committee (protocol #M16-16-028). The study is registered to ClinicalTrials.gov (NCT02915185).

### Statistical analysis

Due to the small number of participants in the LI and MI groups, data from these 2 groups were combined to create a larger LI treatment group. Henceforth, the LI group refers to 8 participants who underwent either LI- or MI-intensity training.

Sociodemographic characteristics between the LI and HI groups were compared using the Mann–Whitney *U* test for continuous variables and χ^2^ tests of independence for proportions. Descriptive statistics were used to characterize the sample at baseline, and the Mann–Whitney *U* test was used again to evaluate between-group differences in outcome measures at baseline. To compare observations between all measurement periods, repeated-measures ANOVAs were conducted for each outcome variable with time (T1 vs. T2 vs. T3) as the repeated factor and training group (LI/MI, HI) as the between-subject factors. Where significant differences were detected between measurement times, post-hoc analysis with Holm-Bonferroni correction was performed to identify between which measurement periods there was a change in the outcome variable. All statistics were computed using R Statistical Software (version 4.0.1) (36).

## RESULTS

### Baseline sociodemographic characteristics and clinical outcomes between groups

Twenty-six participants were reassessed at the 1-year follow-up. Of these, 1 had to be excluded for experiencing a second stroke in the months after completing the intervention. As seen in Table I, participants in the LI and HI training groups shared common characteristics with regard to demographics and history of stroke (i.e. time since stroke, side of stroke, type of stroke). However, participants in the HI training group exhibited significantly higher scores on most clinical measures than those in the LI training group.

**Table I:** Sociodemographic characteristics and clinical outcomes for both training groups [Mean (SD)]

Sociodemographic Characteristics	LI (*n* = 8)	HI (*n* = 17)	*p* *
Age (years)	66 (9)	66 (9)	1.00
Handedness (right/left)	7/1	13/4	0.91
Sex (male/female)	5/3	13/4	0.80
Time since stroke (years)	4 (5)	5 (4)	0.32
Side of stroke (right/left)	4/4	8/9	1.00
Type of stroke (ischemic/hemorrhagic/other)	7/1/0	12/4/1	0.60
**Clinical outcomes**			
FMA (normal = 66)	42 (16)	61 (13)	0.003
BBT (# of blocks in 60 s)	19 (17)	49 (16)	0.003
GS (in kg)	17 (14)	35 (12)	0.006
MAL AOU (normal = 5)	2.04 (1.78)	4.14 (1.45)	0.016
MAL QOU (normal = 5)	1.88 (1.63)	3.83 (1.44)	0.031
Shoulder AROM flexion (°)	117 (47)	147 (37)	0.085
Elbow AROM flexion (°)	138 (7)	138 (9)	0.884
Wrist AROM flexion (°)	39 (23)	67 (21)	0.004

* Mann-Whitney U Test for continuous variables, Chi-square test for independence for proportions; FMA: Fugl-Meyer Assessment; BBT: Box and Block Test; GS: Grip Strength; MAL AOU: Quantitative Motor Activity Log; MAL QOU: Qualitative Motor Activity Log; AROM: Active Range of Motion; LI: low-intensity; HI: high-intensity.

### Differences between assessments over time

A significant effect of time was noted for all outcome measures, except BBT and AROM at the elbow and wrist (see Table II). Also, the 2 training groups improved similarly for most outcome measures, as seen in the lack of significant interaction effects between Training group X Time. However, for the FMA and AROM at the shoulder, post-hoc analysis of the Training group X Time interaction showed that only participants in the LI/MI training group improved from T1 to T2 (FMA: *p* = 0.003; Shoulder: *p* = 0.023) and maintained their gains at the FUP evaluation (FMA: *p* = 0.527; Shoulder: *p* = 1), whereas the HI training group did not show any changes between T1 and T2 (FMA: *p* = 0.527; Shoulder: *p* = 1) and T2 and FUP (FMA: *p* = 0.752; Shoulder: *p* = 1).

**Table II:** Changes in outcome measures over time in both training groups

	Time (F_2, 22_)	*p*	Training group (F_1, 23_)	*p*	Interaction effect (F_2, 22_)	*p*
FMA	22.120	< 0.001^*^	8.147	0.009^*^	10.778	< 0.001^*^
BBT	3.068	0.056	14.020	< 0.001^*^	1.815	0.174
GS	8.999	< 0.001^*^	11.714	0.002^*^	1.283	0.287
MAL AOU	4.941^ɛ^	0.024^ɛ*^	10.602	0.003^*^	0.369^ɛ^	0.615^ɛ^
MAL QOU	20.745^ɛ^	< 0.001^ɛ*^	9.653	< 0.005^*^	0.516^ɛ^	0.538^ɛ^
Shoulder AROM	8.278	< 0.001^*^	2.136	0.157	3.777	0.030^*^
Elbow AROM	0.615	0.545	0.480	0.495	0.562	0.574
Wrist AROM	1.279	0.288	7.882	0.010^*^	1.377	0.263

FMA: Fugl-Meyer Stroke Assessment; GS: Grip strength; BBT: Box and Block test; MAL: Motor Activity Log; AROM: active range of motion.

(*) indicates significance of corresponding *F* test,

(ɛ) indicates Greenhouse-Geisser correction.

## DISCUSSION

To the best of our knowledge, the present study is the first to evaluate the long-term effects of a tailored, MEP-based UE strength training intervention in chronic stroke survivors. On average, participants maintained post-intervention improvements in UE function until at least 1-year follow-up. For most outcome measures, participants’ gains were not modulated by their level of impairment, as measured by their MEP amplitude.

### The long-term efficacy of rehabilitation therapies in individuals with chronic stroke

Our results are in line with the studies that have found long-term benefits of rehabilitation interventions in chronic stroke survivors. Ramos-Murguialday and colleagues showed that, following a 4-week intervention of both Brain-Machine-Interface (BMI) training and physiotherapy, a cohort of individuals with chronic stroke outperformed a control group at a 1-year follow-up as assessed by the FMA (24). The intervention took place every weekday and consisted of 1 h of BMI training, where the participant’s paretic UE was moved by a robotic orthosis, either in response to sensorimotor rhythms (intervention group) or at random (control), followed by an hour of physiotherapy. Sale and colleagues showed that serial robotic training resulted in a long-term improvement in UE function as measured at 1-year follow-up in individuals with chronic stroke, traumatic brain injury, and spinal cord injury (25). Specifically, participants who underwent a 2nd round of robot-assisted therapy, beginning 3 months after the termination of initial treatment, demonstrated improved scores on the BBT and Frenchay Arm Test compared to the control group. Given that robotic training allows for intense training, and that our intervention individualized training according to the person’s own recovery potential to guarantee optimal training intensity, it may be thought that to achieve or maintain post-training gains, intensity plays an important role for chronic stroke individuals.

Although our study did not specifically evaluate participants’ performance using their UE during daily activities, the sustained gains in UE function observed at the 1-year follow-up evaluation are quite promising for everyday UE use. From studies having found that enhanced UE function can lead to improved independence in performing daily tasks such as dressing or toileting (5) as well as allowing for an increased participation in social activities (4), it can be thought that our tailored MEP-based arm training could allow for a greater arm use and better performance in basic and instrumental everyday activities. The ability to use the affected UE more effectively could also boost stroke survivors’ confidence in using it, encouraging a more frequent use and further UE functional gains, as seen even at the chronic stage of a stroke (2).

### The effect of stroke severity on recovery potential

In our study, except for FMA score and shoulder AROM, participants’ maintenance of gains in UE function was not affected by the severity of their stroke, as measured by MEP amplitude. This result contradicts existing literature concerning the question of whether stroke severity modulates recovery potential (13, 27, 37–39). For example, Stinear and colleagues used MEP amplitude and FA of the corticospinal tract (CST) to predict the state of post-stroke participants’ UE function and their functional recovery potential (27). They found that the presence of MEPs modulated the potential for recovery, as those with MEPs could see functional recovery as late as 3 years post-stroke, while recovery in those without MEPs was heavily dependent on damage to the CST. Likewise, Prabhakaran and colleagues modeled the recovery of 41 individuals with acute ischemic stroke of varying severity, as measured by UE FMA score at baseline (38). Clinical variables, including age, sex, lesion location, infarct volume, time between evaluations, and stroke severity, were found to be strong predictors of recovery for only individuals with mild-to-moderate impairment post-stroke; those with severe impairment demonstrated little recovery. Most recently, Bonkhoff and colleagues reaffirmed the distinction between the recovery patterns of individuals with moderate stroke and those with severe stroke (40). Considering those with UE FMA scores less than 45, the authors constructed a Bayesian hierarchical model to predict participants’ change in FMA scores over the period of 6 months. While both the moderate and severe groups were found to experience a similar average change in FMA score over time, it was concluded that individuals with severe stroke-related impairments recovered more the smaller their impairment level was, while for better-recovered stroke survivors, they recovered more the larger their initial impairment (40).

There are several reasons for the discrepancy between the results of our study and those of previous ones. By having tailored our UE strength training intensity to participants’ recovery potential, our intervention may have been uniquely useful in allowing gains in UE function for more severe chronic strokes. In comparison, existing research reflects other, more generic interventions and are thus less effective for recovery from a severe stroke than the intervention used in the present study (13, 27, 38, 40). Also, our study concerns exclusively those in the chronic phase of stroke, and it is possible that the differences in recovery potential between those of mild to severe strokes are attenuated as one moves into the chronic phase of recovery (13, 38, 39). Another reason might be the exclusion criteria of the present study. Individuals presenting significant spasticity or pain intensity at the affected UL, along with a major sensory deficit or hemineglect, were excluded from the study. It is possible that individuals disproportionately contribute to the variation in recovery patterns between severe and less severe stroke survivors, and thus the present study lacks variation between the groups. Finally, in a previous study by Milot and colleagues, where the authors compared the predictive power of fMRI, diffusion-tensor imaging, and MEPs elicited from TMS in predicting UE motor recovery following an 8-week robotic training intervention (37), it was found that MEP magnitude at baseline was the most significant predictor of change in BBT scores between pre- and post-intervention. It was also noted that participants with lower MEP amplitude at baseline experienced greater improvements in BBT scores. The authors attributed this effect to participants having more room to improve with training. It is possible that a similar effect is being observed in the present study for our more severely impacted participants. Further looking at the data, it was noted that in the entire cohort, 3 out of 4 participants that showed a decline in UE function following training were in the HI training group, thus having better recovered from their stroke. That FMA score maintenance over time was modulated by the MEP group; it may be due to the HI group’s higher mean FMA score at baseline (Table I). Indeed, while the maximum score for FMA is 66, the HI group’s mean score was 61, while that of the LI group was only 42. This higher baseline FMA score may introduce a ceiling effect, wherein after post-treatment assessment, participants of the LI group have room to improve their FMA score, while those in the HI group do not. Because of this, we expect the interaction effect between time and impairment level in predicting functional performance to be a feature specific to the FMA, and not functional recovery processes.

Additionally, we found that improvement in AROM in the paretic shoulder, on average, was maintained over time, as opposed to the elbow and wrist range of motion, which saw no improvement. Because of the shoulder’s critical importance in the functional use of the UE (41), this finding further suggests that considering MEP amplitude in the prescription of post-stroke strength training exercises is crucial to optimize short- and long-term training response and recovery in the chronic phase of a stroke. The fact that the shoulder plays a critical role in motor recovery may also underlie the significant interaction effect between Training Group and Time when it comes to predicting shoulder range of motion. Specifically, we found that those in the LI group improved their shoulder range of motion to a greater extent, between T1 and T2, compared to those in the HI group. We suspect that the LI group experienced greater recovery because they had more room to improve (37).

Overall, our results reaffirm that modulating strength training programs by a biomarker of CST integrity leads to short- and long-term UE functional improvements, irrespective of the individual’s initial severity of stroke.

### Study limitations

As for the study limitations, we mentioned previously that FMA scores were high for many participants in the HI training group, which may have introduced a ceiling effect and concealed the subtle improvements in motor impairment otherwise made by these participants. Additionally, because the follow-up study was conducted throughout 2 different sites, potential inconsistencies in data collection may have occurred. However, the research team involved in data collection underwent training before any data was collected to limit this potential problem. The exclusion criteria of the study, which precluded the participation of post-stroke individuals who were unable to perform the training program, limits the generalizability of the results in the population of chronic stroke survivors. Finally, the uneven distribution of participants across treatment intensity groups, in addition to the relatively small sample size of the study, may also be considered a confounding factor.

In conclusion, individuals with chronic stroke whose UE strength training intervention was tailored by a biomarker of corticospinal integrity by means of MEP amplitude saw improvements in functional ability of the UE that were sustained for at least 1 year following the intervention. Moreover, the present study supports the growing body of evidence that long-term functional recovery is a feasible goal for individuals with chronic stroke and suggests that rehabilitation is a worthwhile endeavor for those with more severe stroke impairments.
